# Developing a national strategy of consumer and community involvement (CCI) for women’s health research

**DOI:** 10.1186/s40900-023-00504-8

**Published:** 2023-10-18

**Authors:** Rebecca L. Madill, Leslie D. Arnott, Lesley Pascuzzi, Katie Allen, Angela L. Todd, Janette Perz, Helen Bolger-Harris, Gita D. Mishra, Jacqueline A. Boyle

**Affiliations:** 1 Monash Centre for Health Research and Implementation, Monash University, Clayton, VIC Australia; 2 Consumer Advisor/Advocate, Perth, WA Australia; 3 Consumer Advisor/Advocate, Brisbane, QLD Australia; 4 Faculty of Medicine and Health, The University of Sydney, Camperdown, NSW Australia; 5 Translational Health Research Institute, Western Sydney University, Penrith, NSW Australia; 6 Women’s Health Research Translation and Impact Network, Monash University, Clayton, VIC Australia; 7 School of Public Health, Faculty of Medicine, University of Queensland, Brisbane, QLD Australia; 8 Health Systems and Equity, Eastern Health Clinical School, Monash University, Box Hill, VIC Australia

**Keywords:** Healthcare, Women’s health, Community participation, Patient participation, Health equity

## Abstract

**Objective:**

To develop a consumer and community involvement (CCI) strategy for the Women’s Health Research, Translation and Impact Network (WHRTN), an initiative of the Australian Health Research Alliance (AHRA).

**Type of program:**

A national network, comprising representatives from 14 nationally-accredited research translation centres that aims to embed CCI at a systems level, to improve equity and health outcomes across women’s health.

**Methods:**

A CCI Sub-Committee of WHRTN was established, chaired by a Consumer Advisor/Advocate. This committee invited both internal and external Consumer Advisor/Advocates to participate in a workshop, to guide the development of WHRTN’s CCI Strategy in women’s health research.

**Results:**

A CCI Strategy document was written with input from workshop attendees and leading academics in women’s health and has now been implemented into WHRTN, informing all aspect of the Network’s programs and activities.

**Discussion:**

Broad and early consumer involvement can facilitate meaningful partnerships between researchers and community, and enable genuine consumer contributions to research across strategy development, priority setting and undertaking research. Appropriate finances and time need to be allocated for CCI, with training in CCI a key enabler for its effective implementation.

## Abstract (plain language summary)

Consumer and community involvement in research is increasingly recognised as an important component of high-quality research. It is now required by many research funders and organisations. However, researchers and organisations often struggle with how to initiate and implement consumer and community involvement at a systems level. In this paper, we outline the processes used to develop a national consumer and community involvement strategy for the Australian Health Research Alliance, Women’s Health Research Translation and Impact Network. This provides a roadmap of how organisations can achieve a framework that supports consumer and community involvement across the research pathway. The strategy highlights the need for broad and early inclusion of consumers in decision making, financing consumer involvement, allowing time to build partnerships, and inclusion of training for researchers and consumers.

## Background

The importance and increasing expectation of partnering with consumers in health and medical research in Australia has been outlined by a number of key organisations including the National Health and Medical Research Council (NHRMC) [1], the Australian Health Research Alliance (AHRA) [2] and the Australian Commission on Safety and Quality in Health Care [3]. This is also true for the international context where countries such as Canada [4], the United Kingdom [5] and the United States of America [6], outline increased requirements for patient and public involvement, patient centred research, consumers and/or community representatives to be involved in health and medical research.

Consumers help research to be more community-relevant and meaningful [3] yet embedding Consumer and Community Involvement (CCI) at a systems level to support research remains challenging. Importantly, having consumers who can represent their broader communities is critical. Consumers are people who have a lived experience of a health issue or are people who represent the views and interests of a consumer organisation, a community or a wider constituency [7, 8] Consumer groups often require representation by consumer advisor/advocates to provide a voice, to any concerns they may have about their care [8]. Consumer Advisor/Advocates are individuals who bring ***both*** their personal and communities’ lived experiences and knowledge to research activities, to influence health care outcomes [9, 10].

Here we outline a comprehensive process that has facilitated development of a national strategy with identifiable benefits of CCI including recognition of a shared purpose by researchers and Consumer Advisor/Advocates to affect systems level change. The term Consumer Advisor/Advocate was informed by both Indigenous and non-Indigenous WHRTN consumers who believed this term more accurately reflected the work they provided and the term used by their communities.

### Defining consumer and community involvement

CCI in health and medical research has increased across Australia over the last decade and is a term increasingly used within research to describe the involvement of consumers as partners in research practices, polices and processes [11, 12]. Internationally, terms such as Patient and Public Involvement and Engagement (PPI/E) [13] and Patient Centred Research (PCR) [6] are commonly used.

CCI in health and medical research refers to “the active partnership between researchers, health professionals and those affected by, or who may benefit from, research or healthcare improvement. CCI is about projects being carried out with, or by consumers and community members rather than to, about, or for them.”. [14]p1

The Canadian Institute for Health Research (2020) suggests that CCI builds active and meaningful collaboration with consumers in areas such as governance, priority setting, research and knowledge translation [15]. Harrington and associates (2020) concur and highlight that interaction occurs across all stages of the research process, where research decision making is guided by patient engagement and/or consumer contributions as partners, recognising their specific experiences, values, and expertise [16].

### The Women’s Health Research Translation and Impact Network

The context of developing our CCI Strategy sits within AHRA [17], which has identified the advancement of CCI as a key priority and as part of a national systems level initiative. AHRA is comprised of 14 nationally-accredited research translation centres across Australia. Each research translation centre is comprised of acute health services, community health services, primary care, research institutes, universities and government, with the aim of translating research into best practice to improve patient care and health outcomes for the population [17].

The Women’s Health Research, Translation and Impact Network (WHRTN) is a national network, under the auspices of AHRA, established in 2020 [18]. The Network aims to improve the health of women by integrating prevention, healthcare, research, and translation activities as well as advancing and supporting the careers of women in research [18]. There are nine key areas of women’s health prioritised by the Network [18]. These are:

- Preconception, pregnancy, postpartum and intrapartum health of women and babies
- Mental health
- Reproductive health
- Chronic disease and preventative health, including cancer and heart disease
- Healthy lifestyle, nutrition, physical activity and the prevention of obesity
- Violence and abuse
- Indigenous health^1^
- Healthy ageing
- Sexual health

### WHRTN governance

The WHRTN governance comprises one Steering Committee and four Sub-Committees; Indigenous, Workforce Development, CCI, and Research. The case study for this CCI project sits within the WHRTN CCI Sub-Committee.

During the establishment phase of WHRTN in 2020–21, there were three Consumer Advisor/Advocates, six academic staff and two professional staff on the WHRTN CCI Sub-Committee, with an experienced senior Consumer Advisor/Advocate and academic as co-chairs. All Consumer Advisor/Advocates were remunerated in accordance with Monash Partners’ guidelines [19]. During this phase, a key focus of the CCI Sub-Committee was to integrate an equity and inclusion lens across the Network. Thus, input from extensive and diverse consumers and community was sought to develop a national strategy and its subsequent implementation plan.

### Consumer Advisor/Advocates in the context of WHRTN

Consumer Advisor/Advocates sit at a senior advisory level on all WHRTN executive committees. This includes the Steering Committee and each Sub-Committee. These Consumer Advisor/Advocates were specifically selected because of their previous memberships on health and/or research committees; their high level knowledge of the Australian health system; their representation of diverse communities; and/or their connection into community networks. Collectively they provide WHRTN with both a lived experience and a voice that is representative of a broad community voice.

WHRTN consults with external Consumer Advisor/Advocates who also bring both their lived experience and strong community connections to our tables, providing WHRTN with an even broader range of community perspectives [20].

## Methods

### Developing a national CCI strategy

**Fig. 1:**
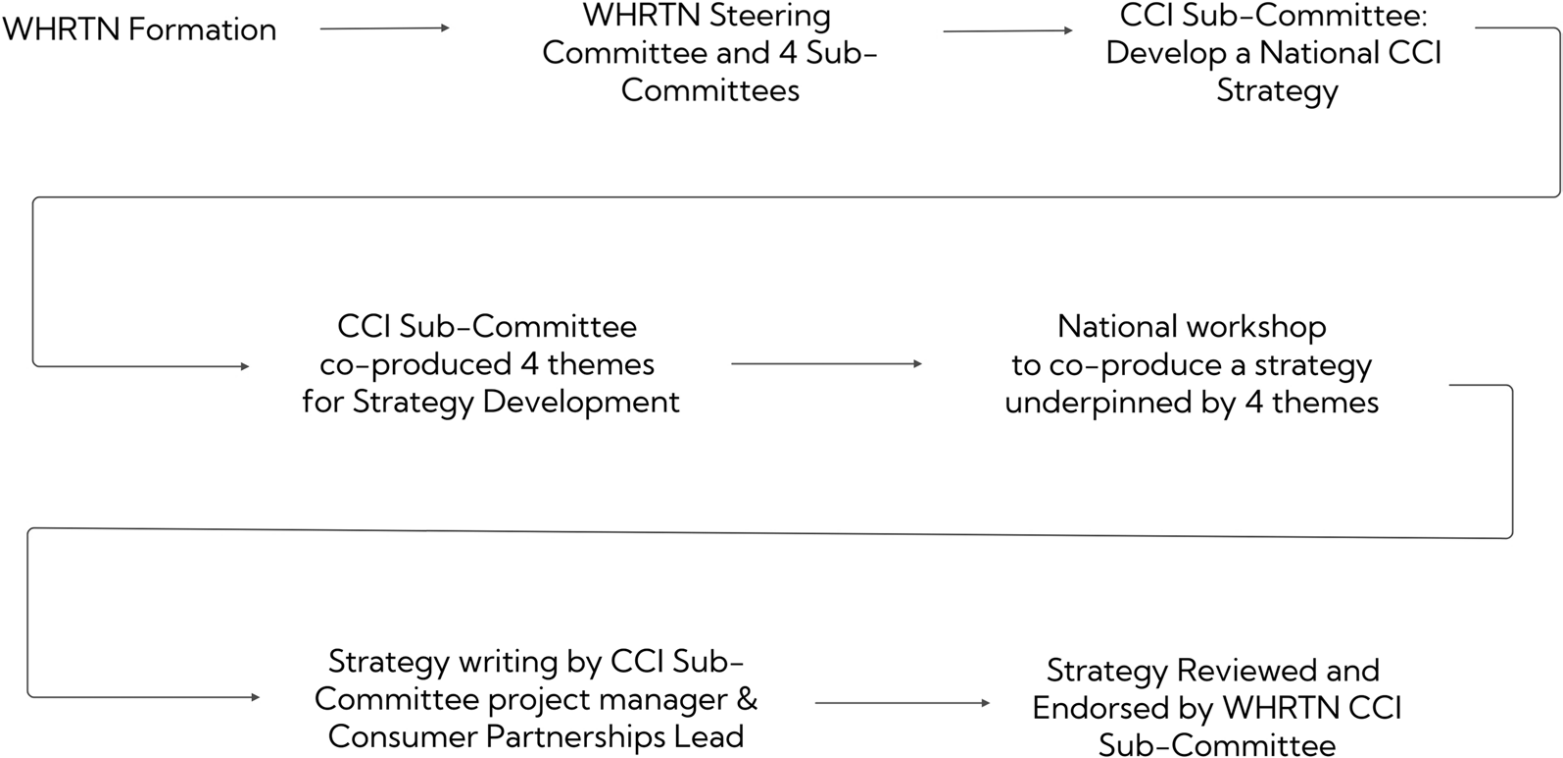
Progression of WHRTN CCI strategy development

The impetus for the overall WHRTN CCI Strategy resulted from WHRTN’s initial funding application that articulated the development of a CCI Strategy. The Strategy development (Fig. 1) was initiated through the CCI Sub-Committee using a co-production process involving Consumer Advisor/Advocates and researchers. Four main themes for Strategy development emerged:

- 1. The development of a National Women’s Health CCI Alliance.
- 2. Connecting and involving women from underserved/marginalized populations.
- 3. Identifying, connecting and involving Consumer Advisor/Advocates.
- 4. Staying connected.

These themes later guided the Strategy development through a Consumer-led workshop.

Consumers were invited to a two-hour, online Consumer-led workshop in June 2021 to develop a framework for the WHRTN CCI Strategy. The aim of the workshop was to capture a shared purpose from diverse consumer groups with a variety of agendas, perspectives and priorities, and to create a vision of how Consumer Advisor/Advocates and researchers, working together, could improve outcomes in women’s health. The Strategy was written by key members of the CCI Sub-Committee, before reviewed and endorsed by all members of the CCI Sub-Committee.

### Identify, connect, invite & organise

Representatives from organisations outside WHRTN were sought to increase community reach. Fifty possible national and state-based Consumer-led and peak body women’s health organisations aligned with WHRTN’s nine health priority areas [16] were identified through personal or professional contacts, snowballing and internet searching. Broad representation was sought including those from culturally and linguistically diverse, Indigenous, gender diverse, and disability backgrounds.

#### Inclusion criteria

All members from our CCI Sub-Committee selected two organisations which aligned closely with each priority area and which had the most impact and reach. The two organisations ranked highest by CCI members from each priority area were then invited to join the workshop (a total of 18). CCI members declared any conflicts of interest when determining which organisations aligned most closely with WHRTN’s 9 priority areas. Organisations were contacted by email, with follow up phone calls and emails. Once each organisation’s representatives were identified, they were contacted to determine their interest in participating, and if so, were invited to attend online meetings to discuss any workshop related questions.

#### Exclusion criteria

Organisations were not included if they did not align with WHRTN’s 9 priority areas and/or if they did not respond to email invitations.

### Workshop planning

The workshop was held online via zoom, where the four main discussion points identified previously (see Sect. "Developing a national CCI strategy") were considered.

Four Consumer Advisor/Advocates from WHRTN led workshop discussions in breakout rooms and WHRTN academic representatives took notes to support the consumer leads. A pre-workshop planning session was held with WHRTN Consumer Advisor/Advocates to discuss key points, answer any questions and address any concerns.

The WHRTN Consumer Advisor/Advocates convened each breakout room and facilitated self-directed discussions with the workshop participants. Each breakout group focussed on two main discussion points before moving onto additional discussion points. The two main discussion points were different for each group to ensure all topics were covered. Researchers from the CCI Sub-Committee provided their time as scribes to document key themes and ideas. They also provided support on an ad hoc basis for any research related questions. Breakout rooms were conducted to create smaller groups, providing a safe space for all participants to be heard. This also allowed for easier facilitation by WHRTN Consumer Advisor/Advocates, who could support some voices to be heard that may have been silent in a larger group.

After breakout room discussions, a whole group discussion using a whiteboarding exercise enabled participants to anonymously contribute their thoughts and ideas. Comments were summarised under the four main discussion points, presented above.

## Results

### The workshop and strategic plan

The workshop participants included 16 consumers from ten consumer-led and/or peak body organisations, four WHRTN Consumer Advisor/Advocates, eight academic members affiliated with WHRTN and two professional WHRTN staff. Feedback was received during the workshop through facilitated group discussion and note-taking through scribes.

Learnings from the workshop included feedback from some WHRTN Consumer Advisor/Advocates who facilitated group discussions in breakout rooms and voiced concerns about how to meet expectations and manage dominant and persistent voices in group discussions. Some consumer participants identified challenges in managing technological requirements and contributing to large groups online. However, overall the workshop feedback was positive and directly informed the WHRTN CCI Strategic Plan, a shared purpose of embedding CCI, and the identification of six high level objectives (Table1). These objectives were equally important, however objectives one to three were actioned prior to items four to six as they were prerequisites for the successful enactment of subsequent objectives. The Strategy was reviewed and endorsed by the WHRTN CCI Sub-Committee and later supported by the development of an Implementation Plan.

**Table 1:** Strategy objectives, actions and activities to implement the strategy for the WHRTN CCI sub-committee, 2021–2024

Strategy Objectives for the WHRTN CCI Sub-Committee, 2021–2024	Actions to Implement the Strategy for the WHRTN CCI Sub-Committee, 2021–2024	Activities to Implement the Strategy for the WHRTN CCI Sub-Committee, 2021–2024
1. Ensure active and ongoing consumer representation and involvement on all WHRTN Committees	1. Recruitment of Consumer Advisor/Advocates to all WHRTN committees	1. Guideline development for Consumer Advisor/Advocate recruitment onto WHRTN committees, including meeting frequency, remuneration, length of appointment and terms of reference
2. Provide CCI capability building and training for WHRTN Consumer Advisor/Advocates and researchers	2. Develop WHRTN CCI capability building and training document disseminated to Consumer Advisor/Advocates and researchers	2. Continue to identify resources to assist with CCI capability building and training for Consumer Advisor/Advocates and researchers
3. Identify and involve Consumer champions/leaders	3. Ongoing identification and involvement of Consumer Leaders	3. WHRTN’s Consumer Partnerships Lead to liaise with Consumer -led networks to assist with the identification of Consumer champions/leaders
4. Engage with underserved populations	4. Engaging with internal and external stakeholders	4. Members from the CCI Sub-Committee will continue to connect with internal and external stakeholders to engage with underserved populations
5. Explore the formation of a National Women's Health CCI Alliance	5. Development of a partnerships agreement and ongoing relationship building with the Australian Women’s Health Alliance & WHRTN	5. Continue to work in partnership with the Australian Women’s Health Alliance & WHRTN and explore CCI collaboration opportunities
6. Enable and support co-production in research	6. Providing support to seed funding grant activities for co-produced research in each WHRTN health priority area	6. Collaborate with the WHRTN Research Sub-Committee to identify processes and develop a framework for co-production seed funding grants

### The implementation plan

The WHRTN Implementation Plan identified outcomes for each of the Strategic Plan actions (Table 1) and initial activities to be undertaken. For example, to support Strategic Objective #1, the following activities were identified: (i) the CCI Sub-Committee chair is to be one of six consumer committee members (equal to academic numbers); (ii) three Consumer Advisor/Advocates are to be appointed to each of the Steering Committee, Research Sub-Committee, Workforce Development Sub-Committee and Indigenous Sub-Committee. It was acknowledged that these numbers would provide support to WHRTN’s Consumer Advisor/Advocates and collectively provide a broad consumer and community perspective.; (iii) Selection of Consumer members on all WHRTN committees were to have a mix of diverse backgrounds to support equity; and (iv) the appointment of a Consumer Partnerships Lead role. This was to be, a specialised role for a senior Consumer Advisor/Advocate, with a focus on liaising/partnering with consumers, community organisations and other relevant stakeholder organisations/groups/individuals as required, including those from underserved populations.

### Implementation of the strategic plan and outcomes

Since the workshop, WHRTN has implemented the majority of the Strategic Plan activities. The Network has appointed the required number of consumers for its committees, providing a good mix of cultural diversity, including Aboriginal and Torres Strait Islander representatives. WHRTN continues to reach out nationally to build our CCI network and increase diversity across gender, sexuality and disability.

To increase capability and capacity-building for consumers, researchers and other healthcare professionals in women’s health, training materials have been developed and disseminated [21]. Additionally, WHRTN’s Consumer Partnerships Lead continues to lead a series of CCI webinars to inform and educate early and mid-career researchers about how to connect and effectively work with women from underserved populations, as partners in research projects [22].

WHRTN has also formalised a partnerships agreement with the Australian Women’s Health Alliance an independent peak and national health promotion charity [23]^.^ This partnership will explore the possibility of establishing a national Women’s Health CCI Alliance.

One of the flagship projects WHRTN has undertaken, led by the inaugural Chair of the Research Committee and the Consumer Partnerships Lead, has been the introduction of co-production grants that provide funding for the development of women’s health research projects. These involve Consumer Advisor/Advocates as research partners, are co-led by a Consumer Advisor/Advocate and senior academic, and have up to 5 academic investigator positions and 5 Consumer Advisor/Advocate positions on each project team. Training in co-production for consumers and researchers has been undertaken by the Consumer Partnerships Lead.

### Planned evaluation

The Strategy has yearly project milestones which track the progression of project activities and outcomes. Additionally, an evaluation of WHRTN is currently underway that includes the experiences of Consumer Advisor/Advocates and researchers to identify enablers and barriers to advancing CCI across the Network. A separate evaluation is planned for the co-production grants in 2024 to capture the uniqueness of their co-productive processes in comparison to traditional research methods and practices.

## Discussion

Our work to develop a national strategy for women’s health research relied on Consumer Advisor/Advocates as key stakeholders in this process. Strong academic support of CCI from WHRTN’s leadership was shown to be a key facilitator for consumer/researcher collaborations which has enabled relationships to flourish in a high-level network such as WHRTN [2]. Financial support, allowing enough time for CCI, and providing resources for CCI, were all found to be fundamental components of successful implementation at a systems level.

To affect ongoing positive systems level change, international and national policy recommendations state the ongoing need for consumers to be involved in shared decision-making processes [24]. In our experience, the appointment of the Consumer Partnerships Lead and a Consumer Advisor/Advocate as chair of the CCI Sub-Committee has been instrumental in facilitating and implementing the WHRTN strategy. This broad and early involvement of Consumer Advisor/Advocates into our CCI strategy development was vital, allowing for early consultation with consumers and stakeholders which has fostered ongoing partnerships and relationships with WHRTN.

Funding which allowed for CCI and the Consumer Partnerships Lead role from the beginning of the WHRTN grant further enabled systems facilitation of CCI. Having Consumer leads involved as early as possible in the research process is also supported by the Patient-Centered Outcomes Research Institute (PCORI), which highly values consumers as shared partners in decision making processes and encourages consumers to be co-investigators in research [25]. Both WHRTN and PCORI recognise the importance of having consumer leaders to authentically reflect consumers’ lived experiences in health and medical research [25, 26].

Whilst funding was a key enabler for successful implementation of consumer involvement for WHRTN, it is recognised that CCI often faces financial barriers [27], and Merner and colleagues (2023) highlight that inadequate funding can constrain partnerships between consumers and organisations [28]. Funding organisations and research policy makers are urged to recognise the cost and time required to support the building of genuine consumer-researcher partnerships. Ensuring sufficient budget in grant applications is recommended to enable and sustain CCI from the inception of the research, to allow for appropriate remuneration for Consumer Advisor/Advocates and the Consumer Partnerships Lead role at all project phases.

Allowing enough time for CCI to develop and relationships to flourish is a significant enabler of successful systems implementation of CCI. Similar to researchers, many consumers have differing and competing priorities and accommodating specific deadlines and/or meeting times is not always possible. Time allows for opportunities to build trust and strengthen relationships with consumers and consumer groups which can lead to more meaningful outcomes [29]. Javanparast and colleagues (2022) also highlight that taking the time to connect with consumers from consumer-led and peak body organisations is vital to consumer stakeholder involvement [27].

For our WHRTN strategy, careful selection of participants allowed for a diversity of experiences and expertise to be present at the consumer-led workshop. We recommend that, ideally, organisations aim for a mix of Consumer Advisor/Advocates who provide a combination of the lived experience (relevant to the research project), a systems knowledge, a connection to community and consumer networks and experience working on committees with various stakeholders including clinicians, researchers and consumers. For our project we chose Consumer Advisor/Advocates without a clinical background (the representatives were not health professionals) to ensure that consumer and community representation remained independent.

Whilst it is recognised that early financial support from WHRTN’s funder was instrumental for advancing CCI, funders’ time constraints can hinder the adaptation of successfully embedding CCI to its full potential. This was the case in this project, as our funding timelines did not allow enough time for external consumer stakeholders to review the WHRTN strategy before endorsement. To strengthen this process in future, adequate time should be allocated to allow for consumer stakeholders’ input at all stages, ensuring their involvement is optimised and accurately captured. WHRTN therefore continues to reflect on ways to ensure sufficient time is allocated to support full consumer involvement at all levels of the research process.

Training of researchers and consumers is seen as another key enabler of CCI [9, 30]. Since our initial CCI training document [21], WHRTN has demonstrated its commitment to CCI training through an ongoing CCI webinar series, with a focus on engaging with underserved populations [22]. WHRTN’s Consumer Partnerships Lead role has been instrumental in the development and delivery of these webinars, facilitating consumer expertise for a wider audience. Ayton et al. (2022) highlight the specific need for researchers to undertake CCI training to build their capacity [30]. McKenzie et al. (2016) support this, stating that researchers who undertook consumer training not only increased their awareness of CCI, but in some instances, it also led to behaviour change through increased consumer involvement in research [9]. Additionally, as our network develops, WHRTN will continue to share CCI resources across AHRA’s research translation centres and other stakeholders. Furthermore, WHRTN is now in the process of exploring a national CCI alliance with the Australian Women’s Health Alliance.

WHRTN acknowledges that overall our CCI Strategy has laid a solid foundation from which CCI continues to develop. Though gaps still exist, our board and early consumer involvement has helped develop a comprehensive Strategic Plan, enabling us to advance consumer involvement across the Network. The development of the WHRTN CCI Strategy and ongoing consumer involvement across the Network continues to be an iteratively adaptive process and this novel approach allows for regular advice and feedback to be sought from consumers. WHRTN supports the need for an integrated systems level approach for CCI with consumers and researchers working in partnerships, to co-produce knowledge [30]. Having consumer leaders in research authentically values the lived experience and moves away from traditional research hierarchies [26]. A further illustration of WHRTN’s commitment to advancing CCI across its network is the involvement of Consumer Advisor/Advocates partnering with research academics to co-produce this body of work.

## Conclusion

Since our initial consumer workshop in June 2021, the WHRTN consumer network continues to grow, involving Consumer Advisor/Advocates and stakeholders from diverse backgrounds. WHRTN continues to demonstrate the importance of embedding CCI in women’s health research to ensure outcomes are meaningful and tangible. We continue to work with our Consumer Advisor/Advocates to co-produce key documents, to co-produce person-centred research, and to continually learn from the end-user perspective. This informs our work, ensuring research is relevant and easily translated into world’s best practice.

## Data Availability

Not applicable.

## Abbreviations

AHRA: Australian health research alliance
CCI: Consumer and community involvement
MRFF: Medical research future fund
NHMRC: National health and medical research council
PCR: Patient centred research
PCORI: Patient-centered outcomes research institute
PPI/E: Patient and public involvement and engagement
WHRTN: Women’s health research translation and impact network

## Footnotes

1 Indigenous Health refers to health for First Nations Aboriginal and/or Torres Strait Islander peoples of Australia.
